# GoLoCo-Net: global-local guided contextual attention network for medical images segmentation

**DOI:** 10.1038/s41598-026-42415-0

**Published:** 2026-03-05

**Authors:** Ying He, Marc E. Miquel, Qianni Zhang

**Affiliations:** 1 School of Electronic Engineering and Computer Science, Queen Mary University of London, London, E1 4NS UK; 2 Clinical Physics, Barts Health NHS Trust, West Smithfield, London, EC1A 7BE UK; 3 Department of Clinical Imaging and Medical Physics, Magnetic Resonance & Non-Ionising RadiationR Physics, Guys and St Thomas’s NHS Foundation Trust, London, SE1 7EH UK; 4 School of Biomedical Engineering and Imaging Sciences, King’s College London, London, SE1 7EH UK

**Keywords:** Computational biology and bioinformatics, Mathematics and computing

## Abstract

Accurate medical image segmentation plays a vital role in assisting diagnosis with quantifiable visual evidence. Due to the complex structure and diverse patterns in medical images, it is crucial to capture both short and long-range pixel relations. While transformers are adept at modeling long-range spatial dependencies in images, they struggle with learning local pixel relationships. To address this, we propose a deep learning network named GoLoCo-Net incorporating a dual decoder structure. More specifically, one decoder entails a Contextual Attention Feature Enhancement (CAFE) module to enhance the features for a broader capture of local and global contexts, whereas the other uses a Global-Guide-Local Feature (GGLF) module that leverages high-level features to enrich low-level features with a global context. The proposed method is evaluated on two dynamic MRI datasets and one multi-organ CT dataset. Experimental results show that the model achieves state-of-the-art performance across all three datasets. The code is available:https://github.com/Yhe9718/GoLoCoNet.

## Introduction

Medical image segmentation plays an integral part in many medical diagnoses (e.g. cardiac function), disease monitoring (e.g. tumour size) and treatment planning (e.g. radiotherapy, surgery). This paper focuses on dynamic MRI and, in particular, speech MRI, with extra validation of the methodology on cardiac MRI data. Dynamic MRI of the vocal tract during speech is increasingly used both in speech science and for clinical reasons (e.g. speech disorders, tongue disease)^1,2^ as it does not use ionising radiation and enables the non-invasive visualization of the vocal tract and the various organs of speech or articulators, in particular, the tongue and soft palate (or velum). Visualizing the shape, size, and position of the vocal tract and articulators, such as the soft palate and tongue, is essential in the field of linguistics but also plays a key clinical role for various conditions. For example, it can help with monitoring tongue motion in patients post-glossectomy^3^ and for assessing the movement of the soft palate and in particular velopharyngeal closure in patients with velopharyngeal insufficiency^4^. For a full overview of speech MRI and its analysis and applications, the readers should refer to the recent reviews and recommendations in the field and in particular acquisition^5,6^, and clinical applications to the velopharynx^7^. Meanwhile, cardiac MR (CMR) is now established as a routine methodology in the diagnosis and follow-up of numerous congenital^8^ and ischemic heart disease^9^. In CMR, dynamic sequences are used for various assessments, in particular ventricular function, flow, and perfusion.

With the increasing availability of labeled medical image datasets, effective deep learning methods have been developed for classification, and subsequently led to significant contributions to other vision related tasks, such as instance segmentation, semantic segmentation, and object detection. In Long et al.^10^, the fully convolutional network (FCN) was first proposed and soon became a common option for image segmentation, leading to a number of deep convolutional models^11^. Later, Ronneberger et al.^12^ introduced U-Net for medical image segmentation. The UNet has an encoder-decoder-like structure. The encoder extracts the contextual features from the image with the pooling operations; the decoder gradually restores the image resolution by upsampling low-resolution feature maps from the deep layer of the backbone. The encoded and decoded feature maps are merged with skip connections to recover further detail. While the UNet performs well for segmenting medical images, its capacity to learn about long-range dependencies between pixels is constrained by the convolutional operation. To address this issue, Attention-UNet integrates the attention mechanism into the UNet architecture by adding an attention gate at each skip connection and thus enhancing the network’s capacity to capture long-range dependencies^13^. Wang proposed a non-local block that can be inserted into intermediate convolutional layers to model the global spatial dependencies of all pixels^14^. These methods, to some extent, improve the CNN’s ability to learn long-range spatial information.

Transformer models were first developed in the field of natural language processing for machine translation^15^, focusing on modeling long-range dependencies with self-attention that can capture correlations between all input tokens. Numerous studies have investigated the transformer’s applicability in computer vision^16^. The Vision Transformer (ViT) was proposed by Dosovitskiy et al. (2020) for classification tasks^17^. ViT splits an image into non-overlapping patches, which are then fed into transformer layers with positional embeddings. Liu et al. (2021) introduced the Swin Transformer, enhancing computing efficiency through shifting windows-based attention^18^. The Pyramid Vision Transformer (PVT) is another hierarchical vision transformer^19^ that utilizes spatial reduction attention to improve computational efficiency. Both the Swin Transformer and PVT are hierarchical, making them suitable as backbones for computer vision tasks. They have proven to be effective for medical image segmentation tasks^20,21^. The great ability of capturing long range dependencies motivates the different network design with transformer, He et al. (2022) proposed a fully transformer network to model the long range dependency with utilization of a spatial pyramid pooling to reduce computational cost^22^.

Although CNNs and Transformers show great results in medical image segmentation tasks, both have limitations. Due to the locality of convolutional operations, CNNs still struggle with modeling long-range information. Transformers have emerged as an alternative to CNNs, demonstrating a strong capability for learning global context. However, they are limited in their localization abilities due to insufficient low-level detail. Crucially, simply combining these architectures via standard skip connections or concatenation is often insufficient to reconcile the semantic discrepancy between such distinct feature representations. High-level features typically suffer from ’attention spread,’ where the focus is diffused across the image, leading to poor boundary definition for small anatomical structures. Conversely, low-level features, while rich in high-frequency texture information, lack semantic awareness and are prone to segmenting background noise or artifacts^23,24^. Therefore, we hypothesize that a strictly hierarchical or cascaded fusion is inadequate. Instead, a targeted dual-pathway strategy is required: one that explicitly reconstructs local context within global features to recover shape fidelity, and another that leverages global semantic guidance to filter and refine noisy local details. This motivation drives the design of our dual-decoder architecture, specifically engineered to bridge this semantic gap. As a result, we introduce a novel network called GoLoCo-Net to overcome these limitations by combining the strength of both CNN and Transformers. GoLoCo-Net comprises two novel modules. The Contextual Attention Feature Enhancement (CAFE) module is designed to enhance multi-scale features. It incorporates a U-shaped module consisting of a series of convolutional layers and pooling operations, serving as the context extractor. Conversely, the Global-Guide-Local Feature (GGLF) module complements the local features with global context, ensuring that fine details are not learned in isolation.

Our contributions are summarized in four aspects as follows:

1. Two modules, namely CAFE and GGLF, are proposed. The CAFE effectively extracts local and global context from the multi-scale features, while GGLF complements the local features with global context, which increases the variety of the local features and allows a more holistic understanding of the image’s context. The two models are designed to address limitations in the CNN and transformer.
2. An integrated framework GoLoCo-Net is developed, integrating the strength of the CAFE and GGLF modules. Without losing generalizability, the CAFE and GGLF modules are designed for easy integration into any medical image segmentation systems.
3. A cascaded feature fusion strategy is introduced that fuses features of multi-scale to effectively fuse their context.
4. The proposed GoLoCo-Net is evaluated on two dynamic Magnetic Resonance Imaging (MRI) datasets (speech and cardiac) and one multi-organ CT dataset. The network establishes a strong baseline on the speech MRI dataset and outperforms other SOTA decoder methods on the ACDC and multi-organ datasets. The experimental results demonstrate that the proposed GoLoCo-Net has strong generalizability.

## Related work

### Traditional image segmentation approaches

Image segmentation plays an important role in many downstream tasks. Traditional image segmentation methods include the watershed transform^25^, mean shift^26^, and region growing schemes^27^ that segment the images based on the groups of pixels’ similarity. Therefore, the methods are not effective in capturing the edge information and the segmentation of highly fine structures.

### Deep learning segmentation methods

Numerous deep learning-based segmentation techniques have been introduced as a result of the success of deep learning approaches in performing computer vision tasks. Fully convolutional network (FCN) is the deep neural network that has been a common solution to image segmentation problems^10^. The model extracts multi-level features and upsamples those features to perform the classification/segmentation tasks. The model is adaptable to many backbones, for example, VGG^28^, ResNet^29^. Rather than directly upsampling features from the layers of backbones, UNet consists of an encoder-decoder structure to upsample the high-level feature maps^12^. The encoder and decoder are symmetric and in between, skip connections are employed to enable the model to recover more feature details. Similar to UNet, Attn-UNet employs a symmetric encoder–decoder structure with skip connections^13^. It further incorporates an attention gate at each skip connection, allowing the model to suppress irrelevant regions. CNNs have significantly advanced segmentation by effectively extracting local features detail; however, their performance is limited by the restricted receptive field. While attention-based models can partially alleviate this limitation, they still remain insufficient. Furthermore, recent advances have expanded into foundation models and efficient supervision paradigms. For instance, Liu et al.^30^ introduced PointSAM, a pointly-supervised Segment Anything Model (SAM) framework. Although applied to remote sensing, such methodologies highlight the growing trend of leveraging flexible prompting and interaction for accurate dense prediction, which parallels challenges in medical image analysis.

### Vision transformer

Transformers have recently attracted interest for their ability in resolving problems related to computer vision. Transformer was initially proposed to solve the machine translation task^15^. The Vision Transformer (ViTs) was first used to classify images on ImageNet by Dosovitskiy et al. (2020)^17^. The ViT splits each input training image into a sequence of tokens with a defined length before position embedding to replace the self-attention method with CNN. After that, the tokens are forwarded to the transformer encoder and then a Multi-layer Perceptron (MLP). Experiment results indicate that a pre-trained ViT model performs competitively with the most advanced CNN models for image classification tasks. However, the ViT has some limitations, according to Yuan et al. (2021)^31^ : $\left( 1 \right)$ The simple tokenization of input images fails to model the important local structure, such as edges and lines among pixels around, leading to inefficient training; $\left( 2 \right)$ and the redundant attention backbone design of ViT results in limited feature richness. Tokens-To-Token Vision Transformer (T2T) was introduced to address such limitations^31^. The model progressively structures the images into tokens by combining the neighbouring tokens into one token, leading to overlapping image data in each token, enabling ViT to model the local structure representation of the image. Meanwhile, the computational complexity is also reduced. As the computational complexity of the ViT is quadratic to the size of the image, it is difficult to process high-resolution images. Swin-Transformer is another model proposed to tackle the computational complexity challenge of ViT^18^. The main concept of the Swin-Transformer is the hierarchical representation; more specifically, the training images are split into small-sized patches and gradually increase the patch size through the merging layers. The layer concatenates the features of 2 $\times$ 2 neighbouring patches, which reduces the number of tokens and enables scale-invariance of the patches. Although the ViTs can perform well on image classification tasks, their application to other prediction tasks is restricted. Wang el al. proposed a new transformer, named Pyramid Vision Transformer (PVT) that overcomes the difficulty of porting ViT to various dense prediction tasks^19^. PVT can also serve as a backbone in various vision tasks by replacing CNN with PVT. The experiment result in Wang et al. (2021)^19^ demonstrates that PVT boosts the performance for many downstream tasks in computer vision. So far, most of the proposed ViTs use the pre-training technique to initialize the training parameters of the models. However, the high performance of the ViTs may be a result of the pre-training on large-size datasets^32^.

### Hybrid ViT-CNN architecture

Hybrid models combining CNNs and ViTs have advanced medical image segmentation by integrating local feature extraction with global context modeling. Unlike SwinUNet^33^, which is entirely ViT-based, TransUNet^34^ employs a ViT encoder to capture long-range dependencies while using a CNN-based decoder to refine spatial details, thus improving the segmentation performance. Rahman et al. introduced a pyramid vision transformer (PVT) to extract multi-scale features. The model integrates cascaded attention modules in the decoder to filter irrelevant features^35^. MERIT improves medical image segmentation by employing a multi-scale hierarchical transformer, which applies self-attention across different window sizes to capture features at multiple scales^36^. Similarly, Jiang et al.^37^ proposed GCIFormer which employs a global context interaction strategy for volumetric medical image segmentation. Their work further validates the necessity of enhancing global dependency modeling to capture complex anatomical structures, a motivation shared by our proposed approach. Moreover, Carion et al. used a CNN for initial feature extraction, followed by a transformer module for refinement^38^. This retains the efficiency of CNNs in local feature extraction while leveraging transformers for long-range dependencies modeling.

Inspired by the success of the vision transformer and the hybrid model, we propose a new network named GoLoCo-Net, employing a pyramid vision transformer encoder to extract multi-level features from images, as well as a UNet-like decoder that progressively recovers image resolution to avoid the gap between high-level features and image resolution. In addition, we present a context extraction module to improve context extraction from encoded features.

### Medical image segmentation

MRI is increasingly used in speech studies. Different methods have been employed to segment the speech-vocal tract. Bresch et al. outline a technique for the unsupervised segmentation of the upper airways^39^. It introduces a segmentation technique that processes a lengthy series of real-time magnetic resonance images using an anatomically informed object model. Silva et al. also introduce an unsupervised segmentation method of the vocal tract for upper airway real-time MRI images based on an active appearance model^40^. As deep learning gains popularity, the convolutional neural network is used to segment the air-tissue boundaries^41–43^. The first work to segment the vocal tract and articulators in speech real-time magnetic resonance images was developed by Ruthven^44^. To segment the speech MRI images, the authors used a FCN and achieved great accuracy in terms of dice coefficient and Hausdorff distance. As a continuation of the work done by Ruthven^44^, Peplinski et al.^45^ trained an FCN network with cropped images around the anatomy of the mouth, and an extensive analysis of the result was carried out. The result shows that by cropping the images to only include the vocal tract increases the accuracy. Moreover, Erattakulangara et al.^46^ implemented stacked transfer learning UNet to segment the vocal tract in dynamic speech MRI which leverages low and mid-level features from open-source medical image datasets.

Cardiac MRI is essential for diagnosing and evaluating various cardiovascular diseases, while multi-organ segmentation plays a crucial role in precise organ localization for diagnosis. Various methods have been proposed for computer-assisted intervention^47,48^, including traditional approaches such as thresholding, clustering, and contour-based techniques. Given the effectiveness of CNNs and ViTs in computer vision tasks, several deep learning models have been developed for cardiac segmentation^34,49^ and multi-organ segmentation^35,50^. Chen et al.^34^ integrated ViT with CNNs for cardiac and multi-organ segmentation, while Mostafijur et al.^51^ employed a ViT with a cascaded attention decoder for segmenting the same dataset.

## Method

### Network overview

**Fig. 1:**
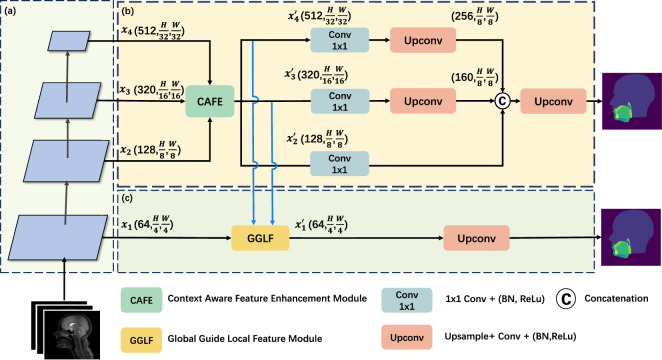
An overview of the GoLoCoNet consisting of three key components: (**a**) a transformer encoder for feature extraction, (**b**) a high-level semantic context decoder branch dedicated to enhancing features with a broader context, which incorporates the Contextual Attention Feature Enhancement (CAFE) module, along with a strategy for gradual upsample feature fusion, and (**c**) a low-level global-guide-local decoder branch, entailing a Global Guided Local Features (GGLF) module, which utilizes high-level features to enhance local features with a global context.

The GoLoCo-Net comprises three main components, as illustrated in Fig. 1: (a) the pyramid vision transformer encoder, (b) a high-level decoder branch and (c) a low-level decoder branch. The vision transformer encoder extracts the features from the input images. The multi-scale features are then fed into two decoder branches: one dedicated to enhancing high-level features with richer contextual information, and the other focused on refining low-level features to preserve appearance details while incorporating global information. In particular, the high-level features are refined by a Context Aware Feature Enhancement (CAFE) module, which involves a context extractor contatining a varied range of receptive fields to complement the high-level features with a broader context. The enhanced features are then upsampled and fused through concatenation and convolution to create a segmentation map. In the low-level branch, a global-guide-local feature (GGLF) module is designed to enhance the low-level features with guidance of the global context. This is achieved using an attention gate and a convolutional attention block to effectively merge and refine the local features, resulting in a detailed segmentation map. In the following section, the encoder, the two decoder branches, and the design of the key modules are further described.

**Fig. 2:**
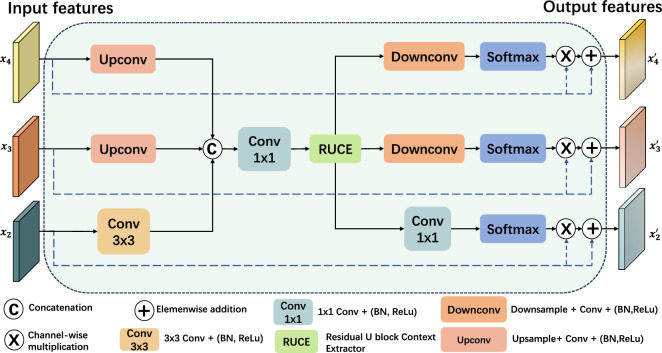
Diagram of the Context-Aware Feature Enhancement (CAFE) module. The module integrates high-level features, which then undergo a Residual U block context extractor (RUCE) module. Subsequently, these enhanced features are combined with the original, unprocessed high-level features to complement more contextual information.

**Fig. 3:**
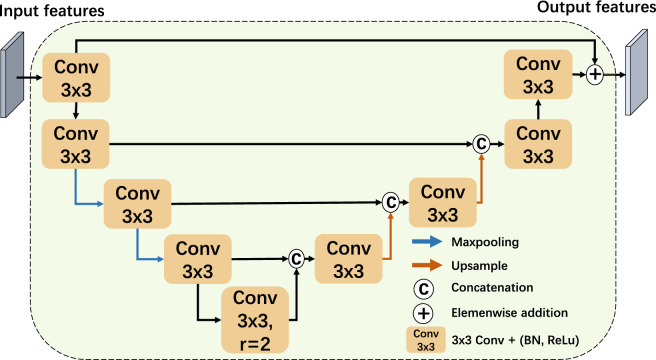
Architecture of the Residual U block Context Extractor (RUCE) module. The module processes a U-shaped structure, comprising a series of pooling operations and consecutive convolutional layers of kernel size three.

**Fig. 4:**
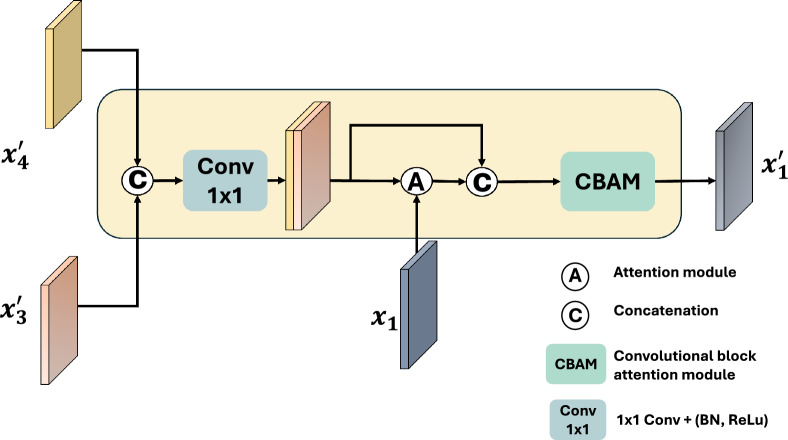
The diagram illustrates the Global Guide Local Feature (GGLF) module’s structure. It utilizes high-level features infused with global context to identify relevant regions using an attention gate, while effectively suppressing the importance of redundant features through a convolutional attention module.

**Fig. 5:**
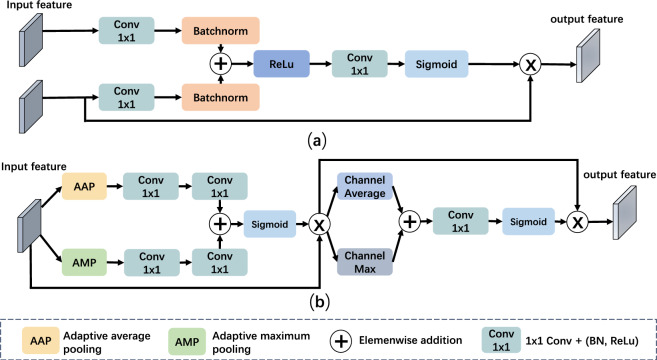
Architecture of GGLF related modules: (**a**) Attention gate module, (**b**) Convolutional block attention module.

### Transformer encoder

The Vision Transformer has demonstrated impressive performance in various vision tasks, with its superior robustness compared to CNNs^52^. The Pyramid Vision Transformer v2 (PVTv2) is a hierarchical variant of the vision transformer that diverges from the conventional approach seen in traditional vision transformers^17,53^. Instead of utilizing a patch embedding module to model spatial information, PVTv2 employs the convolution operation, ensuring consistency in spatial information.

We adopt PVTv2^53^ as the encoder of our proposed model to obtain hierarchical features. The transformer encoder produces multi-scale feature maps, denoted as ${x_1, x_2, x_3, x_4}$, where $x_1$ corresponds to low-level features that primarily capture appearance information, while $x_2$, $x_3$, and $x_4$ represent higher-level features containing rich semantic information.

**Fig. 6:**
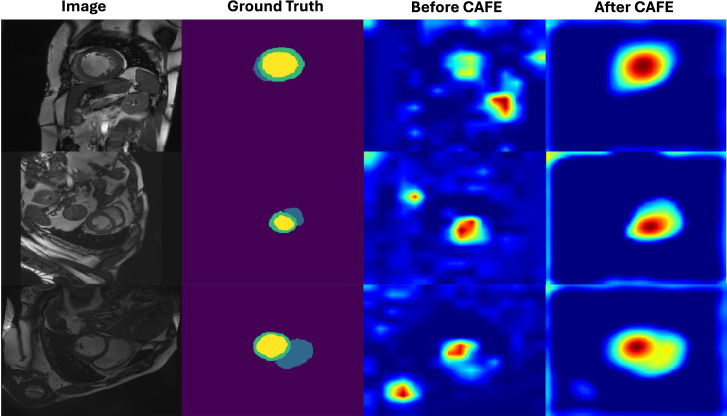
From left to right, an example of ACDC image, the ground truth segmentation of the image, the heat maps of the last encoder layers before and after passing the CAFE module. The heat maps are obtained by adding up feature maps across all channels.

### Contextual attention feature enhancement

As highlighted by Wang et al.^54^, vision transformers have difficulties in recognizing shape and texture attributes due to their inherent limitation in modeling local features. To address this, we introduce dual decoder branches as illustrated in Fig. 1. The branches are specifically designed to overcome the transformer’s limitations in capturing fine-grained local details while enhancing the ability to model broader contextual information at the same time. At the heart of the high-level decoder branch is the Contextual Attention Feature Enhancement (CAFE) module. The module is depicted in Fig. 2. It aims to extract a wider range of contextual information from the high-level encoder layers. The CAFE takes features $x_2$, $x_3$, and $x_4$ from the encoder as inputs.

#### Residual U block context extractor

The residual U block context extractor (RUCE) is illustrated in Fig. 3. The inspiration is drawn from the U2Net architecture^55^, in which residual UNet blocks are stacked with each block utilizing a sequence of downsampling operations to gather multi-scale features. The approach allows for a diverse receptive field and the extraction of a more comprehensive context. In our design, we diverge from the direct stacking of residual U blocks and instead adopt the core design of the residual U block by employing it only once for the extraction of the context from the features to maintain computational efficiency.

The RUCE block has a symmetric encoder–decoder architecture similar to U-Net. It also includes pooling and convolutional layers. The convolutional layers reduce the number of feature channels to ease the computational load. The pooling and upsampling operations decrease and restore the feature resolution, respectively. This process diversifies the receptive field range and enables the extraction of both local and global contextual information from the fused high-level features.

The RUCE block takes an input that is a combination of the multi-scale features from the last three encoder layers. The encoded high-level features, $x_3$ and $x_4$ first passed an upsampling convolutional block to match the resolution $x_2$, while $x_2$ passed through a convolutional layer with kernel size 3. The features are concatenated and then passed through a 1 $\times$ 1 convolutional layer. For feature $x_i$ where $i \in \{2, 3, 4\}$, the input *y* to the RUCE block can be formulated as:

$$\begin{aligned} y = Conv_{1\times 1}([Conv_{3 \times 3}(x_2), Upconv_{3 \times 3}((x_3)), Upconv_{3 \times 3}(x_4)]) \end{aligned}$$

Here, $Conv_{1 \times 1}$ denotes a 1 $\times$ 1 convolutional layer, $[\cdot ]$ represents the concatenation operation and $Upconv_{3 \times 3}$ is defined as a bilinear upsample layer followed by a 3 $\times$ 3 convolutional layer, given by:

$$Upconv_{3 \times 3}(\cdot ) = ReLU(BN(Conv_{3 \times 3}(up(\cdot ))))$$

where $up(\cdot )$ denotes the bilinear upsample layer, $Conv_{3 \times 3}$ is the convolutional layer with a $3 \times 3$ kernel, $BN(\cdot )$ represents the batch normalization function, and $ReLU(\cdot )$ is the ReLU activation function.

As illustrated in Fig. 3, the RUCE block employs pooling functions, convolutional layers and dilated convolutional layers to achieve a varied receptive field for a richer contextual information extraction from the input feature. The first convolutional layer adjusts the input feature’s channel number to match the output channel. The feature is then downsampled twice through the maximum pooling function, with each downsampling followed by a convolutional layer with a kernel size of 3. To maintain computational efficiency, all convolutional layers in the encoder part of the RUCE block after the first convolutional layer preserve the same channel number. Additionally, the bridging layer employs a dilated convolution with a dilation rate of 2 to better capture global information.

The decoder part in the RUCE upsamples the features from the bridge layer to restore feature’s resolution. To effectively recover information lost during the downsampling phase in the encoder, skip connections are used to merge the upsampled and downsampled features. The skip connections, together with the progressive upsampling, are crucial for preserving and incorporating essential information within the module, which allows an enhanced contextual understanding of the feature. Consequently, the output from the last and first convolutional layers is added to combine the enhanced information with the input feature.

#### Context integration

The RUCE output provides enriched contextual information that enhances the encoded high-level features. To integrate contextual information with high-level features, the RUCE output is processed through three parallel branches, each containing either a downsampling convolutional block, denoted as $Downconv(\cdot )$, or a standard convolutional layer. The downsampling convolutional block includes a max-pooling layer followed by a convolutional layer with a $1 \times 1$ kernel, a batch normalization layer, and a ReLU activation function. It is expressed as follows:

$$Downconv_{1 \times 1}(\cdot ) = ReLU(BN(Conv_{1 \times 1}(mp(\cdot ))))$$

where $mp(\cdot )$ is the max-pooling layer, $Conv_{1 \times 1}$ is the convolutional layer with a $1 \times 1$ kernel, $BN(\cdot )$ is the batch normalization function, and $ReLU(\cdot )$ is the ReLU activation function.

Subsequently, three feature maps that correspond to the high-level encoded features are obtained. Each feature is then applied with a softmax activation function, multiplied by the corresponding encoded features of the same dimensions, and added to the corresponding encoded features. Let $x^{\prime }_{i}$ for $i \in$ 2,3,4 be the output from the CAFE module. Let $x_j$ for $j \in \{2, 3, 4\}$ represent the encoded feature from the *j*-th encoder layer, and C denote the context-enhanced feature from the RUCE block. Then, the enhanced feature after CAFE module can be expressed as:

$$x^{\prime }_{2} = (x_2 \times Softmax(Conv_{1\times 1}(C))) + x_2$$

$$x^{\prime }_{3} = (x_{3} \times Softmax(Downconv(C))) + x_{3}$$

$$x^{\prime }_{4} = (x_4 \times Softmax(Downconv(C))) + x_4$$

where Softmax($\cdot$) is a Softmax activation function, and Downconv is the downsampling convolutional block.

To visualize the enhancement by CAFE, we utilize GradCam^56^ to obtain the attention heat map using the cardiac dataset. The attention heatmap from the final layer of the encoder with and without the CAFE is displayed in Fig. 6. It is shown that the RUCE module effectively eases the attention spread.

#### Cascaded feature fusion

Following the CAFE module, the enhanced features pass convolutional layers of kernel 1, and are upsampled to get the segmentation map. Instead of directly upsampling to full resolution, the enhanced features, $x_{3}^{\prime }$ and $x_{4}^{\prime }$, are upsampled to match the resolution of $x_{2}^{\prime }$ with a reduced number of channels by upsampling convolutional blocks, as shown in Fig. 1b. This gradual upsampling strategy helps preserve fine-grained details while ensuring information consistency across features of different scales. The features are then concatenated and passed another upsampling convolutional block to get the segmentation map for the high-level branch. The process can be described as:

$$P = Upconv\left( [x_{2}^{\prime }, Upconv(x_{3}^{\prime }), Upconv(x_{4}^{\prime })]\right)$$

where *P* is the predicted segmentation feature map, [$\cdot$] is the concatenation function, $\text {Upconv}_{1\times 1}$ is the bilinear upsample layer followed by a convolutional layer with a kernel size of $1 \times 1$, and $x_{i}^{\prime }$ for $i \in \{2, 3, 4\}$ are the enhanced feature maps from CAFE.

### Low-level feature enrichment

The shallow layer features from the encoder possess information about the edge and texture detail, but possess little semantic information^57^. In contrast, the deeper encoder layer feature contains rich semantic information^58^. While combining the strengths of both feature levels is intuitively appealing, direct fusion can introduce semantic inconsistencies. To address this, we leverage the semantically enriched high-level features to guide and refine the low-level features.

#### The global guide local feature module

**Fig. 7:**
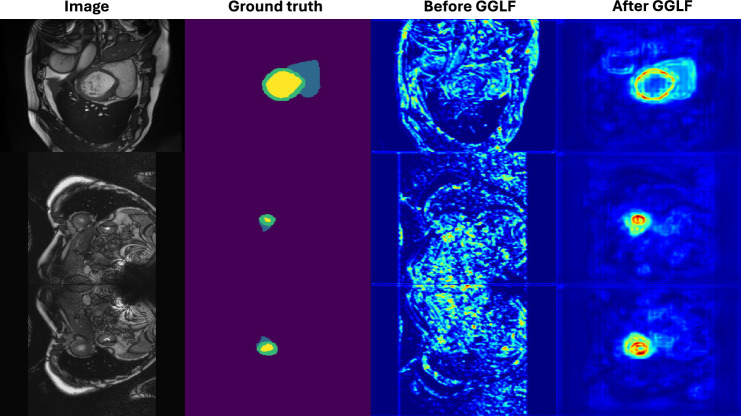
From left to right, an example of ACDC image, the ground truth segmentation of the image, the feature map of the first encoder layers feature before and after passing the GGLF module.

We introduce the Global Guide Local Feature (GGLF) module, which leverages enriched high-level features to guide and refine the shallow layer, $x_1$ with semantic information. The GGLF module is illustrated in Fig. 4. Initially, the rich semantic features from the upper level, $x'_3$ and $x'_4$, are concatenated and passed through a convolutional layer with a kernel size of $1 \times 1$. Subsequently, the combined features and the features from the first encoder layer, $x_1$, are directed into an attention block. The attention block is depicted in Figs. 5a and 6. It efficiently suppresses irrelevant regions in the feature maps by leveraging guidance from high-level features as the feature map shows in Fig. 7. The attention module att($\cdot$) is formulated as:

$$\begin{aligned} q_{att}(h, l) = \sigma _1\left( BN\left( Conv_{h}(h) + BN(Conv_{l}(l))\right) \right) \end{aligned}$$

$$\begin{aligned} \text {att}(h, l) = x \times \sigma _2\left( BN\left( Conv(q_{\text {att}}(h, l))\right) \right) \end{aligned}$$

where $\sigma _1(\cdot )$ and $\sigma _2(\cdot )$ are the Sigmoid activation functions, respectively. $\text {Conv}_{h}(\cdot )$, $\text {Conv}_{x}(\cdot )$, and $\text {Conv}(\cdot )$ are convolutional layers with a kernel size of $1 \times 1$. BN is the batch normalization layer. *h* and *l* are the aggregated high-level features and features from the first encoder layer, respectively.

The output from the attention map is combined with the aggregated high-level features and subjected to a Convolutional Block Attention Module (CBAM)^59^ for additional refinement of the feature maps. As illustrated in Fig. 5b, the CBAM incorporates channel attention (CA), spatial attention (SA) and a convolutional block as expressed in the following:

$$\begin{aligned} CBAM(x) = ConvBlock(SA(CA(f))) \end{aligned}$$

The Channel attention (CA) focuses on recalibrating feature maps based on inter-channel dependencies. It aims to assign different importance weights to different channels, emphasizing relevant channels while suppressing less informative ones. It can be defined as:

1 $$\begin{aligned} CA(x) = \sigma (C_2(\delta (C_1(P_{\text {a}}(x)))) + C_2(\delta (C_1(P_{\text {m}}(x))))) \otimes x \end{aligned}$$

where $\sigma$ is the Sigmoid function. $P_a(\cdot )$ and $P_m(\cdot )$ represent adaptive maximum pooling and adaptive average pooling, respectively. $\delta$ is the ReLU activation function. $C_1$ is the convolutional layer with a kernel size of $1 \times 1$. The convolutional layer, $C_1$ reduces the input feature’s channel dimension by a factor of 16. $C_2$ is another convolutional layer to reduce the feature’s channel number to the number of classes.

The Spatial attention (SA) enables the model to concentrate on relevant regions of the input, allowing for more precise localization of relevant features. It is formulated as:

$$SA(f) = \sigma \left( Conv_{7\times 7}\left( C_m(f) + C_a(x)\right) \right) \times f$$

where $\sigma (\cdot )$ is a Sigmoid activation function. $C_m(\cdot )$ and $C_a(\cdot )$ represent the maximum and average values obtained along the feature map’s channel dimension, respectively. $Conv_{7\times 7}$ is a $7 \times 7$ convolutional layer with padding 3.

The attention module and CBAM work together to suppress irrelevant regions while preserving the contextual information and edge details, which lead to a more accurate segmentation. The enriched features are then upsampled to the full resolution as the input image and passed through a 1 $\times$ 1 convolutional layer to produce the final segmentation map for the low-level branch.

### Joint supervision

In the GoLoCo-Net, the encoder provides hierarchical features which are divided into low-level and high-level branches. The high-level features in the high-level branch are enhanced with a broader context and combined to produce a segmentation map, which can be denoted as $P_1$. The low-level features are complemented with the global context to produce another segmentation map, which can be denoted as $P_2$. These complementary segmentation maps are summed up, and the overall loss can be expressed as:

$$loss = loss_{p_1} + loss_{p_2} + (loss_{p_1 \times p_2})$$

where $loss_{p1}$, $loss_{p2}$ are the supervision losses for the segmented maps $P_1$, $P_2$, respectively. $loss_{p_1 \times p_2}$ is the loss for the multiplicative supervision of $P_1 \times P_2$.

## Experimental setup

### Implementation detail

The implementations of the models used the same Nvidia A5000 graphic card and Pytorch 1.10 to allow a consistent comparison across models. Binary cross entropy (BCE) loss and dice loss were combined as the loss function to train the network. The AdamW optimiser was employed. The hyperparameters for optimization were set to weight decay $=$ 0.00001. The training data batch and validation data batch sizes were set to be 8 and 1, respectively. The learning rate was set to 0.0003. All training images were resized to 256 $\times$ 256 unless otherwise specified. For all baseline models, we followed the default experiment setting as published in their papers. In the testing phase, no post-processing strategies were used. The encoder with pretrained weights on ImageNet^60^ was employed. All implemented experiments were repeated three times, and the result is taken based on the average of the repeated experiments.

### Datasets

**Fig. 8:**
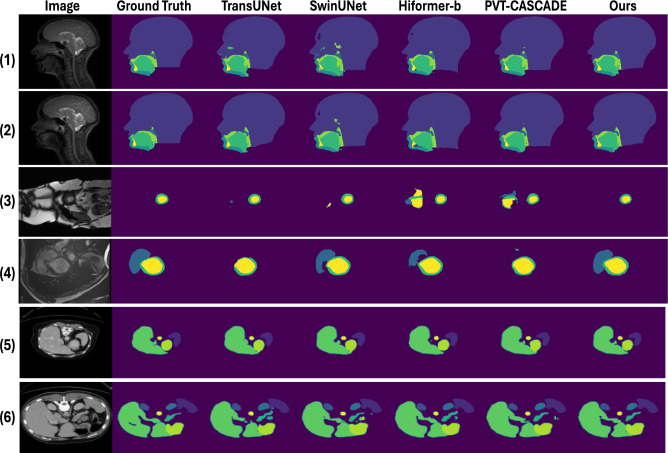
Qualitative comparison of segmentation results across three datasets, demonstrating the effectiveness of our proposed model against existing approaches. Rows 1-2 correspond to the Speech MRI dataset, rows 3-4 to the ACDC dataset, and rows 5-6 to the Synapse dataset. From left to right, the columns display the input image, ground truth, and segmentation results by TransUNet, SwinUNet, Hiformer, PVT-CASCADE, and our proposed network.

**Speech MRI dataset**^61^ The dataset contains five subjects of magnetic resonance images. The series has 105,71,71,78, and 67 images, respectively, as well as the corresponding ground truth. Each image includes regions of six classes as shown in the first and second row of Fig. 8 , namely, the head, jaw, soft-palate, tongue, tooth-space, and vocal-tract. Considering the similarity in neighboring frames within the same sequence, we perform a five-fold cross-validation on the dataset so that for each fold, the sequence for a different subject is used as the testing set.

**ACDC dataset**^62^ The ACDC dataset consists of 100 cardiac MRI scans of different patients. Each scan has three classes, which are right ventricle (RV), left ventricle (LV) and stomach myocardium (Myo). Following MT-UNet^63^, 70 cases (1304 axial slices) are used for training, 10 cases (182 axial slices) for validation, and 20 cases for testing.

**Synapse (Multi-organ CT) dataset**^64^ The Synapse dataset is used for abdominal organ segmentation. It contains 30 abdominal CT scans, comprising 3,779 axial contrast enhanced slices. Each CT scan consists of 85 to 198 slices. For our experiments, we follow the same preprocessing procedure and data split as TransUNet^34^, using 18 scans for training and 12 scans for validation. We segment only eight abdominal organs: the aorta, gallbladder (GB), left kidney (KL), right kidney (KR), liver, pancreas (PC), spleen (SP), and stomach (SM)

### Result evaluation

**Table 1:** Performance comparison of various models on Speech MRI.

Model	#Param (M) $\downarrow$	#FLOPs (G) $\downarrow$	#fps $\uparrow$	Speech MRI						Mean
				Head	Jaw	Soft-palate	Tongue	Tooth-space	Vocal-tract	
UNet^12^	34	65.53	52	99.02	96.86	96.66	98.23	96.03	**97.05**	97.31
TransUNet^34^	105.32	38.36	26	99.46	96.65	96.66	98.31	95.53	96.70	97.20
SwinUNet^33^	27.17	6.20	-	98.51	89.22	89.26	96.27	96.84	92.93	93.84
Hiformer^65^	25.51	38.36	98	98.94	96.57	96.57	98.13	95.06	95.31	96.76
PVT-CASCADE^35^	34.13	7.62	133	99.22	97.18	97.54	98.51	95.60	96.32	97.40
PVT-GCASCADE^66^	26.64	4.25	160	99.50	97.36	**97.56**	98.77	95.80	95.65	97.44
GoLoCo-Net (Proposed)	30.07	8.68	121	**99.63**	**98.07**	97.54	**99.02**	**96.96**	97.00	**98.04**

The bold values indicates the best performing experiment for each model.

The Dice coefficients in $\%$ are reported. For the reported FLOPs, input images of size 224 $\times$ 224 are used for SwinUNet and Hiformer, while input images of size 256 $\times$ 256 are used for the rest models.

**Table 2:** Comparison of the proposed method to other state-of-the-art models on the ACDC dataset.

Model	AvgDICE	RV	M_yo	LV
R50+UNet^34^	87.55	87.10	80.63	94.92
R50+AttnUNet^34^	86.75	87.58	79.20	93.47
nnUNet^34^	91.61	90.24	89.24	95.36
ViT+CUP^34^	81.45	81.46	70.71	92.18
TransUNet^34^	89.71	86.67	87.27	95.18
SwinUNet^33^	88.07	85.77	84.42	94.03
TransCASCADE^35^	91.63	90.25	89.14	95.50
PVT-GCASCADE^66^	91.95	90.31	89.63	95.91
GoLoCo-Net (Proposed)	**92.75**	**92.03**	**90.20**	**96.02**

The bold values indicates the best performing experiment for each model.

The result is reported in Dice score (%).

**Table 3:** Quantitative result of different model’s segmentation performance on Synapse Multi-organ dataset.

Model	Aerage DICE	Aorta	GB	KL	KR	Liver	PC	SP	SM
R50+UNet^34^	74.68	84.18	62.84	79.19	71.29	93.35	48.23	84.41	73.92
R50+AttnUNet^34^	75.57	55.92	63.91	79.20	72.71	93.56	49.37	87.19	74.95
SSFormer^67^	78.01	82.78	63.74	80.72	78.11	93.53	61.53	87.07	76.61
MissFormer^68^	81.96	86.99	68.65	85.21	82.00	94.41	65.67	**91.92**	80.81
TransUNet^34^	77.61	86.56	60.43	80.54	78.53	94.33	58.47	87.06	75.00
SwinUNet^33^	77.58	81.76	65.95	82.32	79.22	93.73	53.81	88.04	75.79
HiFormer^65^	80.39	86.21	65.69	85.23	79.77	94.61	59.52	90.99	81.08
PVT-CASCADE^35^	81.06	83.01	70.59	82.23	80.37	94.08	64.43	90.10	**83.69**
GoLoCo-Net (Proposed)	**83.22**	**88.20**	**72.86**	**88.15**	**83.35**	**95.49**	**66.62**	89.56	81.52

The bold values indicates the best performing experiment for each model.

Dice scores in (%) is presented for each of the class.

**Table 4:** Ablation study comparing the performance of the proposed model on the Speech, ACDC, and Synapse datasets in Dice score (dual branch and multiplicative supervision).

Experiment	Module used		Dataset		
	CAFE	GGLF	Speech	ACDC	Synapse
1	$\checkmark$		97.50	92.00	81.76
2		$\checkmark$	97.20	92.10	82.37
3	$\checkmark$	$\checkmark$	**98.04**	**92.75**	**83.22**

The bold values indicates the best performing experiment for each model.

**Table 5:** Ablation study comparing the performance of the model without the joint multiplicative supervision term in Dice score (dual-branch supervision only).

Experiment	Module used		Dataset		
	CAFE	GGLF	Speech	ACDC	Synapse
1	$\checkmark$		97.14	91.71	81.67
2		$\checkmark$	96.88	91.67	81.83
3	$\checkmark$	$\checkmark$	**97.80**	**92.16**	**82.71**

The bold values indicates the best performing experiment for each model.

**Fig. 9:**
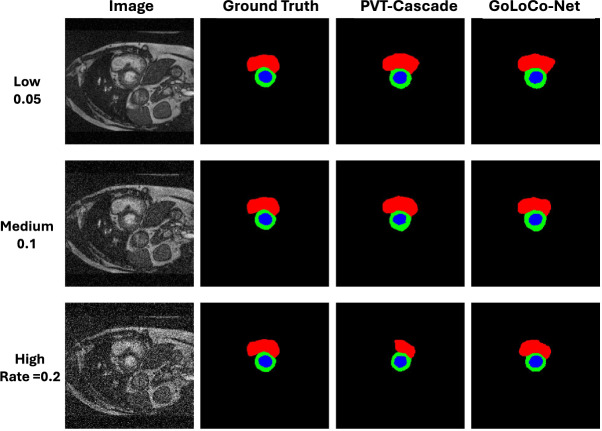
Qualitative robustness comparison on the ACDC dataset. The columns represent (left to right): the input image with added Gaussian noise, the Ground Truth, and segmentation outputs from PVT-Cascade and GoLoco-Net. Rows 1–3 illustrate the effect of increasing Gaussian noise levels (0.05, 0.1, and 0.2, respectively).

#### Result on speech MRI dataset

For a fair comparison, we implemented baseline models that include: UNet^12^, Attention-UNet^13^, and TransUNet^34^, PVT-CASCADE^35^, and PVT-GCASCADE^66^. UNet, Attention-UNet, and TransUNet are frequently used models for the segmentation of medical images. PVT-CASCADE is a recent model that uses a cascaded attention module in the decoder to segment medical images, and PVT-GCASCADE utilizes graph convolutional layer to achieve a lightweight decoding architecture while maintaining accurate segmentation performance. The computational efficiency (parameters, FLOPs, fps) and performance of the different models are given in Table 1. It is shown that our proposed model outperforms the other models in terms of overall mean dice coefficient. In particular, the GoLoCo-Net obtains a mean Dice of 98.04$\%$, which is 0.6$\%$ higher than the second best, and obtained the lowest Hausdorff distance of 2.84. In particular, the Dice score for jaw and soft-palate segmentation is shifted by 1.4$\%$ and 1$\%$, respectively, in comparison to TransUNet. The first two rows of Fig. 8 show qualitative segmentation results for speech MRI dataset. It demonstrates that GoLoCo-Net produces cleaner segmentations with reduced noise while better preserving the anatomical structures of target articulatory classes.

#### Result on ACDC dataset

In addition to the speech MRI dataset, the model was also tested and evaluated on the widely used ACDC dataset, which consists of dynamic MRI scans of a different organ, the heart. The dataset is widely used in medical image segmentation research, serving as a benchmark to evaluate the most competitive methods. The results of GoLoCo-Net in comparison with the state of the art models are presented in Table 2. The GoLoCo-Net achieves an average mean Dice coefficient of $92.75\%$, outperforming the other CNN and transformer based models. The class-specific results indicate that GoLoCo-Net achieves the best performance on right ventricle (RV) and myocardium (Myo) segmentation, and the second best for the left ventricle (LV), with only a marginal difference from the best result. The third and fourth rows of Fig. 8 present the qualitative comparisons between our proposed model and other state-of-the-art models. The results illustrate that our model exhibits superior ability in recognizing the right ventricle, as observed in the fourth row. In addition, it demonstrates an improved ability to preserve the anatomical shape more accurately, as shown in the third row. These highlight the effectiveness of our proposed model.

### Result on Synapse dataset

To assess the generalizability of GoLoCo-Net, we further evaluated the model on a dataset of different imaging modalities. Specifically, we used a multi-class CT (Synapse) dataset to test the model’s performance. Table 3 presents the quantitative results of different models on the Synapse dataset. The results indicate that our proposed model achieves the highest average Dice coefficient of 83.22$\%$, which outperforms other models. Notably, the model attains the best Dice scores in six out of eight classes, demonstrating superior segmentation performance. Furthermore, our model predicts the structure of different classes more consistently than other models, which can be observed from the last two rows of Fig. 8.

## Ablation study

To evaluate the effectiveness of individual components in GoLoCo-Net, we conducted ablation studies examining the contributions of the two proposed modules: CAFE and GGLF.

As presented in Table 4, Model 1, which includes the CAFE but excludes GGLF, achieves Dice scores of 97.50$\%$ on the Speech dataset, 92.00$\%$ on ACDC, and 81.76$\%$ on Synapse. Model 2, containing only the GGLF module, performs similarly to Model 1, with a 0.1$\%$ higher Dice score on ACDC but 0.3$\%$ lower on the Speech task. However, it outperforms Model 1 in Synapse, suggesting that expanding the receptive field with CAFE improves the learning of complex semantic features. Model 3, integrating both CAFE and GGLF, achieves the highest Dice coefficient in all data sets, demonstrating the effectiveness of combining both components for the overall performance of the model. Furthermore, the effectiveness of the third loss term based on the product of the model’s two branches, was investigated. Excluding the multiplicative term in the supervision loss resulted in a slight degradation of the results (Table 5 vs Table 4).

## Conclusion

In this paper, we propose GoLoCo-Net. The model is coupled with a contextual attention feature enhancement (CAFE) and global guide local feature (GGLF) modules to address medical image segmentation by exploiting broader contextual information for low and high-level features. The CAFE module enriches the high-level encoded feature with a broader range of context by introducing a context extractor. The GGLF module leverages enriched high-level features to complement low-level features with global context, leading to a more diverse range of feature context in the local feature branch. The overall experimental results on the Speech MRI, ACDC, and Synapse datasets demonstrate that the proposed model achieves state-of-art performance and exhibits strong generalizability across diverse imaging modalities.

The experimental Speech MRI dataset inherently contains artifacts, in particular off-resonance artifacts observed when the soft palate is elevated and were not corrected for in the acquisition, and motion blurring around the tongue caused by the lower frame rate used which is insufficient to capture the quickest tongue movements. Our results demonstrate the model’s resilience to these artifacts. Furthermore, we evaluated the model’s robustness by introducing Gaussian noise during inference, as illustrated in Fig. 9. Despite the combination of natural artifacts and simulated noise, GoLoco-Net maintained its performance. When compared to state-of-the-art methods, our model demonstrated superiority in identifying targeted classes, even at high noise levels, confirming its robustness.

## Data Availability

The datasets used in this study are publicly available. The speech MRI dataset is at DOI: 10.1038/s41597-023-02766-z, the ACDC dataset at DOI: 10.1109/TMI.2018.2837502, and the Synapse dataset at DOI: 10.7303/SYN3193805.
